# Comparing the Diagnostic Performance of 3D T1-Volumetric Interpolated Breath-Hold Examination (VIBE) MR-Based Pseudo-CT Versus Conventional CT for Detecting and Characterizing Radiographically Occult Hip and Pelvis Fractures

**DOI:** 10.7759/cureus.100237

**Published:** 2025-12-28

**Authors:** Kwan Kit Chan, Chiu Wang Jeffrey Chan, King Kenneth Cheung

**Affiliations:** 1 Department of Radiology, North District Hospital, Hong Kong, HKG; 2 Department of Imaging and Interventional Radiology, Prince of Wales Hospital, Hong Kong, HKG; 3 Department of Imaging and Interventional Radiology, Faculty of Medicine, The Chinese University of Hong Kong, Hong Kong, HKG

**Keywords:** hip fractures, mri, occult fractures, pelvic fractures, pseudo-ct, t1-vibe

## Abstract

Background

Radiographically occult hip and pelvic fractures are relatively common in older adults after low-energy trauma, and delayed or missed diagnosis is associated with worse outcomes. MRI is the preferred modality for detection, while CT remains important for fracture delineation, surgical planning, and use in patients with MRI contraindications. However, using both modalities increases cost, imaging time, and strain on imaging services. MRI-based pseudo-CT (pCT) methods derived from 3D T1-volumetric interpolated breath-hold examination (VIBE) data have shown promise in other settings, but their role in evaluating occult hip and pelvic fractures has not yet been explored. This study aimed to explore the clinical performance and usability of MR-based pCT for detecting and characterizing radiographically occult hip and pelvic fractures. Specifically, the study aimed to compare pCT and conventional CT in terms of fracture detection, fracture characterization, diagnostic confidence, and interobserver agreement.

Methodology

This retrospective study included 22 patients (18 men, 4 women; mean age = 75.7 ± 10.6 years) with a total of 12 radiographically occult hip and pelvic fractures. All patients underwent the same-day MRI and CT imaging, including 3D T1-VIBE sequences processed with grayscale inversion to generate pCT images. Two radiologists independently evaluated both imaging modalities in a blinded and randomized fashion for fracture presence, location, and characteristics. Diagnostic confidence was assessed using a five-point scale. The reference standard was established by two senior radiologists using all available clinical and imaging data. Statistical analyses were performed to evaluate diagnostic performance, diagnostic confidence, and interobserver agreement.

Results

pCT showed diagnostic performance not inferior to CT for both readers (R1 and R2) (predefined non-inferiority Δ = −10%; R1 Δ = +18.2% (95% confidence interval (CI) = −3.7% to +18.2%), p = 0.0026; R2 Δ = +27.3% (95% CI = 2.2% to +27.3%), p = 0.0010). pCT detected all CT-apparent fractures and showed perfect concordance with CT in fracture localization and characterization. There was no statistically significant difference in diagnostic performance between pCT and CT, although pCT for both readers showed a trend toward higher sensitivity (pCT vs. CT; R1 90.0% vs. 60.0%; R2 100% vs. 60.0%), lower specificity (pCT vs. CT; R1 91.7% vs. 100%; R2 83.3% vs. 100%), and higher accuracy (pCT vs. CT; R1 90.9% vs. 81.8%; R2 90.2% vs. 81.8%). There was no significant difference in diagnostic confidence between the two modalities, with pCT demonstrating a trend toward slightly higher confidence when ruling in fractures and slightly lower confidence when ruling out. Interobserver agreement was perfect for CT (κ = 1.00) and moderate for pCT (κ = 0.55).

Conclusions

Compared with conventional CT, 3D T1-VIBE MR-based pCT demonstrated diagnostic performance that was not inferior for detecting radiographically occult hip and pelvic fractures, with perfect concordance in fracture characterization. pCT enables comprehensive fracture assessment within a single MRI examination, and our findings suggest that pCT has the potential to serve as a viable alternative to CT, enabling a “one-stop-shop” imaging approach in appropriate clinical scenarios.

## Introduction

Radiographically occult hip and pelvic fractures represent a critical diagnostic challenge in emergency and orthopedic medicine, occurring in 2-14% of elderly patients after low-energy trauma [1]. Accurate diagnosis is crucial, as missed hip and pelvic geriatric fractures may lead to delayed or absent treatment, which, in turn, is associated with a steep increase in mortality rates, reported to be as high as 46.1% at one year, compared to 18% in those treated operatively [2].

Current evidence consistently supports MRI as the superior modality for detecting occult hip fractures, demonstrating a pooled sensitivity of up to 94%, which surpasses that of conventional CT, with reported figures of 79-92% [3,4]. This is due to MRI’s ability to detect more subtle, often non-displaced fractures by identifying non-structural changes such as increased marrow edema and hemorrhage, which may be inconspicuous on conventional CT, given its reliance on detecting structural bony changes such as cortical disruption or fracture lines. Nonetheless, CT may still be utilized in selected cases, for example, in patients with MRI contraindications, or when further evaluation of fracture lines is required by orthopedic surgeons for surgical decision-making and planning. This often necessitates separate MRI and CT examinations, translating into increased cost, patient transfer time, and demand on imaging resources.

Recently, MRI-based pseudo-CT (pCT) or synthetic-CT (sCT) techniques derived from specialized MR sequences, such as 3D T1-volumetric interpolated breath-hold examination (VIBE), zero echo time (ZTE) imaging, and deep-learning-based algorithms, have shown increasing promise in generating CT-like images from MRI data [5,6]. Clinically, CT-like bone imaging from MRI has been validated across a growing range of applications, offering a radiation-free alternative to conventional CT. These include assessment and longitudinal monitoring of structural bone changes such as erosions, sclerosis, and ankylosis in inflammatory conditions (e.g., sacroiliitis and axial spondyloarthritis) [7]; accurate electron density mapping and dose calculation for MRI-only radiotherapy planning [8]; evaluation of pars interarticularis defects and stress injuries in young and athletic populations [9]; and morphologic assessment of the spine, pelvis, and hip for preoperative planning [10]. Several studies have demonstrated good to excellent agreement between MRI-derived CT-like images and conventional CT for fracture detection, cortical bone delineation, and 3D morphologic measurements, supporting the feasibility of MRI-based alternatives to CT in selected musculoskeletal settings [11].

T1-weighted 3D gradient-echo sequences, such as VIBE, represent a practical approach for generating MRI-based pCT images with CT-like bone contrast. Using short repetition and echo times and optimized flip angles, VIBE provides high-resolution isotropic datasets that, after grayscale inversion, enable CT-like visualization of cortical bone and fracture morphology, with reported good agreement with conventional CT for musculoskeletal applications [12].

By extension, VIBE-based pCT may serve as a suitable technique to replace CT in detecting and characterizing occult hip and pelvic fractures, although to our knowledge, such an application has not previously been investigated. In this study, we aimed to compare the non-inferiority, diagnostic performance, diagnostic confidence, and interobserver agreement of pCT versus conventional CT in detecting and characterizing radiographically occult hip and pelvic fractures.

## Materials and methods

All procedures and imaging performed in this study were conducted in accordance with local institutional ethical standards.

Inclusion criteria

Between June 2024 and June 2025, patients presenting with suspected radiographically occult hip or pelvic fractures (i.e., negative initial radiographs) underwent imaging according to our standard occult fracture protocol. This protocol included plain CT and conventional large field-of-view coronal T1-weighted and T2 fat-suppressed MRI. During this period, an additional MRI-based pCT sequence was incorporated on an opportunistic basis in a subset of patients as part of an institutional pilot implementation.

Selection for the additional pCT sequence was not based on clinical characteristics, imaging findings, or outcomes, but instead depended on operational factors such as scanner availability, scheduling logistics, and feasibility at the time of imaging, thereby minimizing systematic selection bias. All patients who underwent the additional pCT MRI sequence during the study period were included in this retrospective analysis. For included patients, CT and MRI examinations were performed sequentially on the same day in accordance with institutional protocol.

MRI parameters

MRI examinations were performed using a 1.5 T MR scanner (MAGNETOM Sola, Siemens Healthineers). All MRI examinations included a large coronal field-of-view acquisition covering the entire bony pelvis, sacrum, hips, and both proximal femora, using standard 2D T1-weighted and short tau inversion recovery sequences, as well as an additional 3D T1-VIBE sequence (full acquisition parameters are provided in Table 1). Grayscale inversion was applied to the 3D T1-VIBE images to generate the pCT images.

**Table 1:** Summary of MRI parameters. VIBE: volumetric interpolated breath-hold examination; TSE: turbo spin echo; STIR: short tau inversion recovery; TR: repetition time; TE: echo time; FOV: field of view

3D T1-VIBE coronal pelvis protocol	
TR (ms)	16.9
TE (ms)	4.77
Flip angle (^o^)	15
FOV (mm)	336 x 333
Matrix size	444 x 448
Slice thickness (mm)	1.5
Interslice gap (mm)	0.3
2D T1 TSE coronal pelvis protocol	
TR (ms)	460
TE (ms)	8.2
Flip angle (^o^)	150
FOV (mm)	390 x 260
Matrix size	720 x 480
Slice thickness (mm)	4
Interslice gap (mm)	0.4
2D T2 TSE STIR coronal pelvis protocol	
TR (ms)	2960
TE (ms)	49
TI (ms)	160
FOV (mm)	390 x 260
Matrix size	624 x 416
Slice thickness (mm)	4
Interslice gap (mm)	0.4

CT imaging parameters

All CT examinations were performed using a 128-slice SOMATOM Drive CT system (Siemens Healthineers) with CARE Dose 4D automatic exposure control. Anatomical scan coverage matched that of MRI. Imaging parameters were as follows: 120 kV, 280 mAs, one-second rotation time, and 1 mm slice thickness. Images were reconstructed using a B60s kernel and evaluated for analysis.

Data analysis

The pCT and CT images were anonymized, unpaired, and randomly presented to two radiologists, each with approximately two years of post-Fellowship of the Royal College of Radiologists (FRCR) fellowship experience in musculoskeletal imaging at the time of image interpretation. Readers were blinded to all clinical information and all other imaging data. The readers were allowed to analyze the raw images, generate multiplanar reformats, and comment on the presence or absence of fracture, and the location, pattern, and type of fracture.

The reference standard for acute fracture diagnosis was established by retrospective consensus assessment performed by two senior musculoskeletal radiologists, each with more than 10 years of subspecialty experience. This assessment incorporated all available clinical information, including patient history, mechanism of injury, clinical follow-up, and all imaging data, comprising plain radiographs, conventional CT, standard MRI sequences (T1-weighted and T2 fat-suppressed images), the pCT images, and any subsequent follow-up imaging when available. Discrepancies were resolved by consensus discussion, and the final reference diagnosis was used as the ground truth for all analyses.

For the presence or absence of fracture, readers recorded their findings and corresponding diagnostic confidence using a five-point Likert scale, where 1 represented definitely no fracture, and 5 represented definite fracture. For dichotomization, scores of 1 and 2 were considered negative for fracture, while scores of 3 to 5 were classified as positive.

If a fracture was deemed present, readers were required to comment on fracture location and characteristics according to the AO/OTA fracture classification for the pelvic ring, acetabulum, and femur [13].

Statistical analysis

Data from pCT and CT were evaluated and compared on a per-patient, paired basis. For diagnostic performance, sensitivity, specificity, and accuracy of pCT and CT relative to the reference standard were calculated for each reader using contingency tables, along with their corresponding exact Clopper-Pearson 95% confidence intervals (CIs). Paired comparisons between pCT and CT were performed using the McNemar test, with statistical significance defined as α < 0.05. Diagnostic confidence levels between pCT and CT were compared using the Mann-Whitney U test, with statistical significance defined as α < 0.05. For non-inferiority testing, an exact McNemar-type non-inferiority test was employed. Exact (Clopper-Pearson) confidence intervals were calculated for discordant pairs, and pCT was declared non-inferior if the lower limit of the CI exceeded the predefined non-inferiority margin (Δ = −10%) and α < 0.025. Interobserver agreement for each modality was assessed using weighted Cohen’s kappa statistics. Agreement levels were interpreted as follows: κ ≤ 0.20 poor, 0.21-0.40 fair, 0.41-0.60 moderate, 0.61-0.80 substantial, and ≥0.81 almost perfect agreement. Statistical analyses were conducted using MedCalc Statistical Software (version 23.4.5; MedCalc Software Ltd., Ostend, Belgium).

## Results

Study population

**Figure 1:**
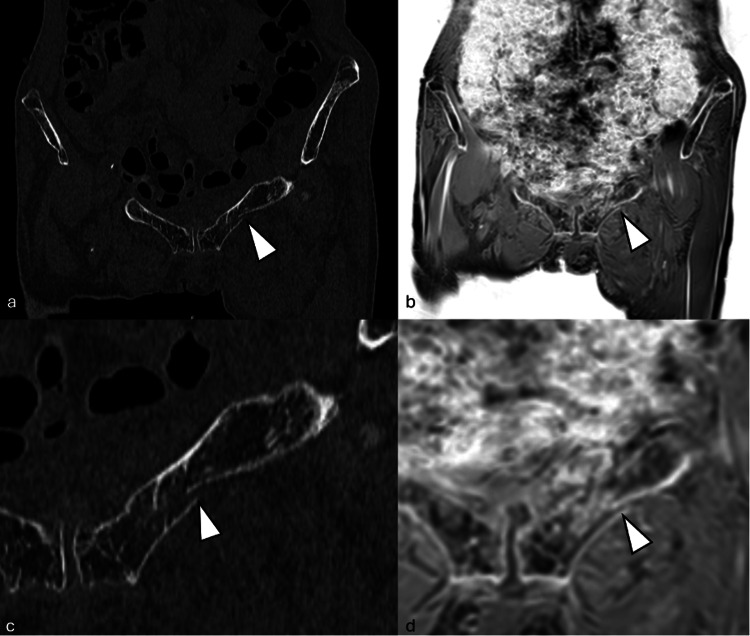
Representative imaging example 1. A patient with a left superior pubic ramus fracture is shown in (a, c) CT and in (b, d) pseudo-CT. The cortical break was clearly visualized and with high resemblance on both modalities (arrowheads).

**Figure 2:**
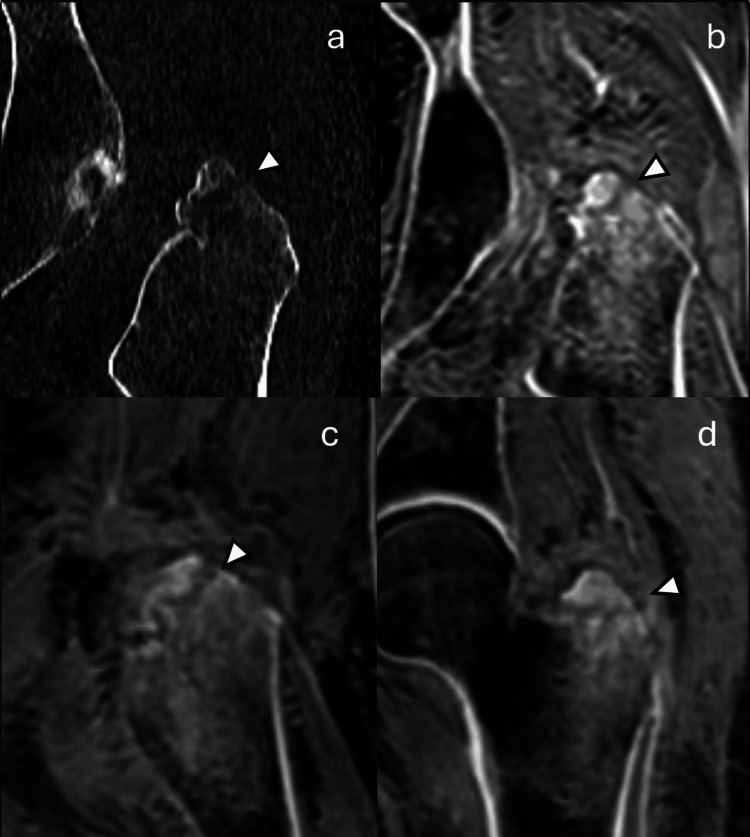
Representative imaging example 2. A patient with a left greater trochanter fracture (arrowheads) seen on (a) CT and (b) pseudo-CT. As with CT, pseudo-CT also allows multiplanar reformatting for more comprehensive fracture visualization and assessment (c, d).

**Figure 3:**
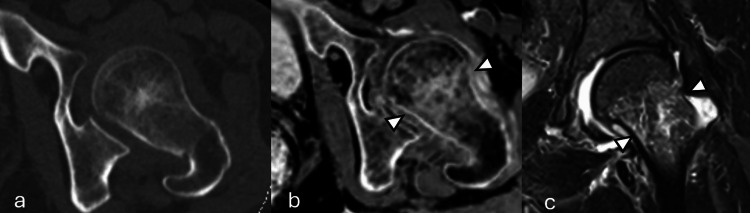
Representative imaging example 3. A patient with a non-displaced left subcapital femoral neck fracture (arrowheads) on (a) axial CT, (b) axial pseudo-CT, and (c) coronal short tau inversion recovery MR. The fracture was not visualized on CT but was conspicuous on pseudo-CT.

**Figure 4:**
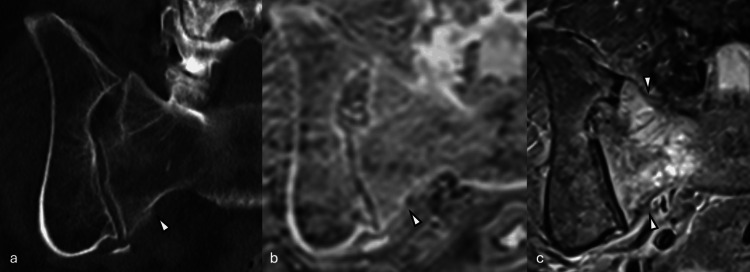
Representative imaging example 4. A patient with a minimally displaced right sacral ala fracture (arrowheads) on (a) coronal CT, (b) coronal pseudo-CT and (c) coronal short tau inversion recovery MR. This case was correctly identified by one of the readers on pseudo-CT but missed by both readers in CT.

**Figure 5:**
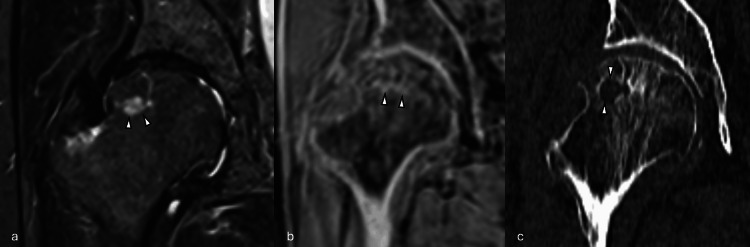
Representative imaging example 5. A false-positive case by pseudo-CT. (a) short tau inversion recovery MR, (b) pseudo-CT, and (c) CT. An intraosseous cyst (arrowheads) at the right femoral head was incorrectly interpreted on pseudo-CT as a fracture by one of the readers.

In total, 24 patients met the inclusion criteria, with two patients excluded due to non-diagnostic image sets from severe motion artefact. A total of 22 patients were ultimately included in the study, including 18 men and four women with a mean age ± 1 SD of 75.7 ± 10.6 years old (range = 64-93 years). According to the reference standard, 12 patients had no fractures, and 10 patients had a total of 12 fractures, which included six femoral, four pelvic (all at the pubic rami), and two sacral fractures. Representative imaging examples are shown in Figures 1-5.

Comparison between pCT and CT against the reference standard for detecting occult fractures

Overall diagnostic performance results are summarized in Table 2. No statistically significant differences were observed between pCT and CT for either reader. However, pCT consistently demonstrated a trend toward higher sensitivity (R1 = 90.0%, R2 = 100%) and higher accuracy (R1 = 90.9%, R2 = 90.2%) compared with CT (both readers: sensitivity = 60.0%, accuracy = 81.8%), at the expense of lower specificity (pCT R1 = 91.7%, R2 = 83.3% vs. CT 100% for both readers).

**Table 2:** Diagnostic performance of pseudo-CT and CT in detecting radiographically occult hip and pelvis fractures within 22 patients. Numbers in brackets are 95% confidence intervals.

Reader	Diagnostic performance	Pseudo-CT (%)	CT (%)	P-value
R1	Sensitivity	90.0 (55.5–99.8)	60.0 (26.2–87.8)	0.248
	Specificity	91.7 (61.5–99.8)	100 (73.5–100)	1.000
	Accuracy	90.9 (70.9–98.9)	81.8 (59.7–94.8)	0.617
R2	Sensitivity	100 (69.2–100)	60.0 (26.2–87.8)	0.134
	Specificity	83.3 (51.6–97.9)	100 (73.5–100)	0.248
	Accuracy	90.2 (70.8–98.9)	81.8 (59.7–94.8)	1.000

Regarding diagnostic confidence, there was a non-significant trend toward slightly higher confidence with pCT when diagnosing the presence of fracture (mean Likert score: R1 = 3.80, R2 = 3.90) compared with CT (R1 = 3.50, R2 = 3.30). Conversely, confidence when diagnosing the absence of fracture was marginally lower with pCT (R1 = 1.33, R2 = 1.83) compared with CT (R1 = 1.25, R2 = 1.33) (Table 3).

**Table 3:** Mean confidence level and standard deviation of pseudo-CT and CT for each reader in detecting radiographically occult hip and pelvis fractures compared to reference standard. Note: Confidence level was evaluated on a five-point Likert scale: 1 = definite no fracture; and 5 = definite fracture. Lesions rated with a confidence level of 1 and 2 were considered negative for fractures, while those rated at confidence levels 3 or more were classified as positive for fractures.

Reader	Fracture by reference standard	Pseudo-CT	CT	U-statistic	P-value
R1	Yes	3.80 ± 1.32	3.50 ± 1.61	28.0	0.089
	No	1.33 ± 0.65	1.25 ± 0.45	91.0	0.215
R2	Yes	3.90 ± 1.10	3.30 ± 1.85	29.5	0.116
	No	1.83 ± 1.27	1.33 ± 0.48	71.5	1.000

Non-inferiority test of pCT against CT for detecting occult fractures

pCT met the predefined non-inferiority criterion relative to CT for both readers. For reader 1, the paired difference was +18.2% (95% CI = −3.7% to +18.2%; exact one-sided p = 0.0026), with the lower bound exceeding the non-inferiority margin of −10%. For reader 2, the paired difference was +27.3% (95% CI = +2.2% to +27.3%; exact one-sided p = 0.0010), likewise demonstrating non-inferiority.

Interobserver agreement

For pCT, the overall observed percentage of agreement (P₀) was 77.3%, and the expected percentage of agreement by chance (Pₑ) was 49.2%. For CT, the overall observed percentage of agreement (P₀) was 100.0%, and the expected percentage of agreement by chance (Pₑ) was 60.3%. pCT showed moderate agreement between readers (kappa = 0.55, 95% CI = 0.22-0.88), while CT showed perfect agreement (kappa = 1.00, 95% CI = 1.00-1.00).

Agreement of fracture characteristics between pCT and CT

For all CT-apparent fracture cases, there was perfect concordance of fracture characterization by pCT in terms of location, pattern, and extension.

## Discussion

In this study, to our knowledge, the first to utilize a 3D T1-VIBE derived pCT sequence for detecting and characterizing radiographically occult hip and pelvic fractures, we demonstrated that pCT shows diagnostic performance that is not inferior to conventional CT, offering accuracy and diagnostic confidence that are at least comparable, while leveraging several of MRI’s intrinsic advantages.

Recent advances in multi-echo time (multi-TE) MR bone imaging have enabled the clinical deployment of sequences using short repetition times (TR) and echo times (TE), optimized flip angles, and versatile T1-weighted 3D-GRE acquisitions. One such sequence is the VIBE, a 3D T1-GRE technique optimized with asymmetric k-space sampling and partial Fourier interpolation to reduce scan times [14]. Similar to conventional CT, VIBE MRI provides strong signal contrast between cortical bone and adjacent soft tissues, facilitating improved visualization of osseous anatomy and accurate assessment of bone pathology in a radiation-free manner [15,16]. These properties have driven growing interest in VIBE-based pCT approaches [17]. While early studies have demonstrated promise for pCT in applications such as radiotherapy planning and chronic bone disease [18,19], its role in acute trauma and occult fracture detection has not previously been explored.

Our findings demonstrate that pCT achieved non-inferior diagnostic performance compared with CT for detecting radiographically occult hip and pelvic fractures. Notably, pCT showed a consistent, albeit non-significant, trend toward higher overall accuracy than CT for both readers. All fractures identified on CT were also detected on pCT, with perfect concordance between the two modalities in characterizing fracture location, extent, and AO/OTA classification.

This observation is clinically significant, as it suggests that a single MRI examination incorporating a VIBE-based pCT sequence could provide both the high sensitivity of conventional MRI and the detailed structural information traditionally obtained from CT. Such an approach has the potential to streamline diagnostic workflows, eliminate the need for additional CT imaging, reduce radiation exposure, and improve overall efficiency in the assessment of suspected occult fractures.

Crucially, pCT demonstrated perfect concordance with CT in fracture characterization, including fracture location, extent, and classification. This is likely attributable to pCT’s ability to generate high-resolution isotropic 3D datasets with strong contrast between cortical bone and surrounding tissues, further enhanced by the ability to perform meaningful multiplanar reconstructions for improved visualization and surgical planning [20], as illustrated in Figure 2.

This concordance in fracture characterization is particularly relevant for clinical decision-making and preoperative planning. It implies that an MRI examination augmented with a pCT sequence could provide all necessary diagnostic and planning information that would otherwise require a separate CT scan, enabling a “one-stop-shop” imaging strategy in appropriate clinical scenarios. Practically, this could reduce radiation exposure, minimize delays, and lower costs associated with patient transfer and repeat imaging.

Although not statistically significant, pCT demonstrated a trend toward higher sensitivity than CT for both readers (90-100% vs 60%), detecting up to four additional fractures that were not identified on CT. Conversely, CT demonstrated 100% specificity, whereas pCT showed slightly lower specificity (83-92%), attributable to three false-positive cases, a predictable trade-off given the higher sensitivity of MRI-based techniques.

These findings reflect the complementary strengths and limitations of MRI and CT. The higher sensitivity of pCT is likely related to MRI’s ability to detect non-structural fracture-related changes, such as bone marrow edema and hemorrhage, in addition to pCT’s capacity to visualize structural cortical abnormalities [19,21]. In contrast, conventional CT relies almost exclusively on identifying structural changes, such as cortical disruption or fracture gaps, and is unable to detect intramedullary signal changes [16]. In this context, dual-energy CT has emerged as a useful adjunct, demonstrating improved sensitivity compared with conventional CT alone and approaching the performance of MRI in some settings [22].

CT’s superior specificity reflects its strength in confirming fractures only when definitive structural changes are present. However, this comes at the cost of sensitivity, as several true fractures identified by pCT were missed on CT. This observation is consistent with prior literature reporting high specificity but comparatively lower sensitivity for CT in the evaluation of occult fractures [3].

A review of discrepant cases suggested that pCT false-positive findings may be partially attributable to motion artefact, a known limitation of MRI, particularly in elderly or unwell patients. Additionally, certain confounding factors, such as normal tissue interfaces or blood products, may mimic cortical bone on inverted grayscale pCT images, leading to occasional overcalling [23,24].

Despite no statistically significant difference in diagnostic confidence between pCT and CT, several notable trends were observed. When diagnosing the presence of fracture, readers demonstrated slightly higher confidence with pCT, potentially reflecting the additional supportive MRI features, such as bone marrow edema and intramedullary signal changes. Conversely, confidence in excluding fractures was marginally lower with pCT, possibly due to increased background signal variability and MRI’s greater susceptibility to artefacts and mimicking signal changes. CT, by comparison, is generally less affected by background noise or confounding signals. Overall, diagnostic confidence with pCT was deemed sufficient and comparable to CT, supporting its operational feasibility.

Interobserver agreement was acceptable for both modalities. CT demonstrated perfect agreement between readers (κ = 1.00), whereas pCT demonstrated moderate agreement (κ = 0.55). The lower agreement observed with pCT may reflect its noisier appearance and greater susceptibility to artefacts and mimics, which can increase interpretative variability. Nevertheless, overall reproducibility remained acceptable, and when considered alongside comparable diagnostic confidence and accuracy, pCT appears interpretable with a reasonable level of consistency in clinical practice.

Taken together, these findings support the role of pCT as a valuable adjunct in the assessment of occult fractures, enabling a streamlined, MRI-only “one-stop-shop” approach that can improve workflow efficiency while avoiding ionizing radiation.

Importantly, the proposed pCT technique is widely accessible and resource-efficient. The 3D T1-VIBE sequence is available as a standard acquisition on Siemens MR systems, with equivalent sequences offered by other vendors, such as THRIVE (Philips) and FSPGR (GE). The additional scan time is short, averaging approximately 3.5 minutes, and pCT images can be generated via simple grayscale inversion, a function readily available on most PACS systems. Interpretation does not require additional hardware, software, or specialized training.

Nonetheless, it is acknowledged that conventional CT remains more widely available and generally less costly than MRI in many emergency and trauma settings. As such, some institutions may continue to favor combined MRI ± CT pathways. However, pCT offers a compelling alternative in scenarios where CT is not feasible or desirable, such as during CT system downtime, when radiation avoidance is prioritized, or in MRI-only environments (e.g., mobile MRI units or standalone imaging centers).

As an MRI-based technique, pCT inherits several limitations of MRI, including contraindications such as claustrophobia and incompatible implants, as well as susceptibility to motion and metal-related artefacts, which may be more pronounced than on CT. Additionally, due to the intrinsic T1-weighted gradient-echo nature of VIBE, materials other than mineralized bone that are also T1-hypointense (e.g., calcification, air, or blood products) may confound interpretation [23]. In such cases, CT may remain a valuable problem-solving modality.

Strengths and limitations

This study has several strengths. First, it employed a paired, within-patient design, allowing direct comparison between pCT and conventional CT while minimizing inter-patient variability related to fracture type, injury mechanism, and patient characteristics. Second, CT and MRI examinations were performed sequentially on the same day using standardized institutional protocols, reducing temporal bias. Third, this study represents one of the first clinical evaluations of VIBE-derived MRI-based pCT for radiographically occult hip and pelvic fractures, providing important preliminary evidence to guide future research.

Several limitations should be acknowledged. First, the small sample size (22 patients) limits statistical power and generalizability. This study was designed as a preliminary exploratory investigation, and no formal a priori power calculation was performed. Consequently, the study may be underpowered to detect small differences between modalities. Nevertheless, the findings serve as a valuable proof-of-concept, demonstrating technical feasibility and potential clinical utility, and provide justification for larger prospective studies.

Second, image interpretation was performed by two radiologists with approximately two years of post-FRCR fellowship experience in musculoskeletal imaging. While this level of experience may be considered limited, it reflects real-world clinical practice, where acute trauma imaging is frequently interpreted by radiologists at similar stages of training. Therefore, the results are likely representative of routine clinical performance.

Third, the VIBE-based pCT technique evaluated in this study does not represent the most advanced pCT or sCT methodologies available. Emerging approaches using ultra-short or zero echo time imaging and deep-learning-based post-processing may offer improved robustness and reduced susceptibility to artefacts. However, compared with these techniques, VIBE-based pCT is substantially more accessible and easily implementable, enhancing its relevance to everyday clinical practice.

## Conclusions

Three-dimensional T1-VIBE MRI-based pCT demonstrates diagnostic performance that is not inferior to conventional CT for detecting radiographically occult hip and pelvic fractures, and shows perfect concordance with CT for fracture characterization. Compared with CT, pCT exhibited a non-significant trend toward higher sensitivity and accuracy, albeit with slightly lower specificity. Although interobserver agreement was lower for pCT, diagnostic confidence remained comparable between modalities. These findings suggest that pCT represents a viable, radiation-free alternative to conventional CT, enabling comprehensive fracture detection and characterization within a single MRI examination. In clinical scenarios where an MRI-only approach is preferred or required, pCT may facilitate a streamlined, efficient, and diagnostically robust imaging pathway for patients with suspected radiographically occult hip and pelvic fractures.
